# Prp16 enables efficient splicing of introns with diverse exonic consensus elements in the short-intron rich *Cryptococcus neoformans* transcriptome

**DOI:** 10.1080/15476286.2025.2477844

**Published:** 2025-03-10

**Authors:** Manendra Singh Negi, Vishnu Priya Krishnan, Niharika Saraf, Usha Vijayraghavan

**Affiliations:** Department of Microbiology and Cell Biology, Indian Institute of Science, Bangalore, India

**Keywords:** *Cryptococcus neoformans*, Prp16, pre-mRNA splicing factor, short and ultra-short intron recognition, exonic consensus elements

## Abstract

DEAH box splicing helicase Prp16 in budding yeast governs spliceosomal remodelling from the branching conformation (C complex) to the exon ligation conformation (C* complex). In this study, we examined the genome-wide functions of Prp16 in the short intron-rich genome of the basidiomycete yeast *Cryptococcus neoformans*. The presence of multiple introns per transcript with intronic features that are more similar to those of higher eukaryotes makes it a promising model for studying spliceosomal splicing. Using a promoter-shutdown conditional Prp16 knockdown strain, we uncovered genome-wide but substrate-specific roles in *C. neoformans* splicing. The splicing functions of Prp16 are dependent on helicase motifs I and II, which are conserved motifs for helicase activity. A small subset of introns spliced independent of Prp16 activity was investigated to discover that exonic sequences at the 5’ splice site (5’SS) and 3’ splice site (3’SS) with stronger affinity for U5 loop 1 are a common feature in these introns. Furthermore, short (60–100nts) and ultrashort introns (<60nts) prevalent in the *C. neoformans* transcriptome were more sensitive to Prp16 knockdown than longer introns, indicating that Prp16 is required for the efficient splicing of short and ultrashort introns. We propose that stronger U5 snRNA-pre-mRNA interactions enable efficient transition of the spliceosome from the first to the second catalytic confirmation in Prp16 knockdown, particularly for short introns and introns with suboptimal features. This study provides insights into fine-tuning spliceosomal helicase function with variations in *cis-*element features.

## Introduction

The removal of introns and splicing of exons of RNA pol II transcribed pre-mRNA is ubiquitous in eukaryotic gene expression. This process is executed by a versatile multi-megadalton complex composed of five snRNPs (U1, U2, U4, U5, and U6) and over 150 proteins known as spliceosomes [1]. *In vitro* splicing assays have been the key to deciphering the stepwise assembly of spliceosomal components in model yeast/human pre-mRNAs. The systematic assembly by conformational and compositional remodelling ensures two sequential transesterification reactions: first, branching leads to a lariat intermediate followed by exon ligation [2]. The characteristic conserved *cis* sequences at the 5’ splice site (5’SS), branch point sequence (BPS), and 3“ splice site (3’SS) and their interaction with spliceosomal snRNAs guide spliceosome assembly, activation, and catalysis [3]. Compositional and conformational remodeling is facilitated by eight ATP-dependent RNA helicases that disrupt or translocate various RNA-protein, RNA-RNA, and protein-protein interactions [4]. During *in vitro* spliceosome assembly, budding yeast DEAD-box helicases, Sub2/UAP56, Prp5, and Prp28, ensure accurate splice site recognition to form the B complex. The Brr2 helicase unwinds the U4/U6 duplex to release U4 during the activation of the B complex, which is followed by the recruitment of NTC to form the B^act^ complex. DEAH box helicase Prp2 remodels the B^act^ complex to the catalytically active B* complex wherein the first catalytic reaction generates the intermediates 5’exon and lariat intron-3’exon held in the C complex. The Prp16 DEAH box helicase remodels the C complex by destabilizing the binding of branching factors, such as Cwc25, and allows the recruitment of exon ligation factors, such as Slu7 and Prp18, thus forming C* complex primed for exon ligation. The second catalytic reaction ligates the 5’ and 3’ exons and excises the lariat introns. The release of spliced mRNA from the post-spliceosomal (P) complex is mediated by Prp22-helicase, whereas Prp43-helicase mediates intron lariat spliceosome (ILS) disassembly [5].

A suppressor mutant screen of *cis* intronic invariant branch nucleotides A to C mutation in a budding yeast splicing reporter minigene identified Prp16 [6,7] as an important factor for branch recognition. Other studies with mutants with diminished ATPase activity have also shown aberrant use of pre-mRNAs with mutant branch nucleotide C. Thus, it was inferred that the ATPase activity of Prp16 ensures splicing fidelity [8]. Subsequent *in vitro* splicing assays showed an important role of *S. cerevisiae* Prp16-mediated proofreading of the substrate before the first step of catalysis [9]. Prp16 mutants defective for ATPase activity stabilized the interaction of first step splicing factors Yju2 and Cwc25 with the mutated substrate, thereby supporting progression to form the branched lariat intron-3’exon. However, the ATPase activity of Prp16 destabilizes branching factors by remodeling the spliceosome after 1^st^ step of catalysis to facilitate exon ligation [10,11]. This dual function of Prp16 leads to a hypothesis where the ATPase activity of Prp16 competes with its ATP-independent facilitation of 5’SS cleavage at the first step to proofread slow branching reaction [12].

The *in vivo* genome-wide relevance of Prp16-mediated spliceosome remodelling in the global transcriptome has not been extensively explored. In fission yeast, Prp16 is required for the splicing of a vast majority of introns in its genome, where its action facilitates first-step catalysis by destabilizing 5’SS-U6snRNA and BS-U2snRNA interactions [13]. Furthermore, *Sp*Prp16 affects fission yeast cell cycle progression and heterochromatinization of centromeres and telomeres [13,14]. In many species with transcriptomes enriched with introns, the diversity of the *cis*-elements and their interactions with the spliceosomal factor machinery can play a critical role in splicing efficiency. Thus, these interactions and the kinetics of splicing progression add an additional layer of complexity to the dynamic regulation of gene expression [15]. This raises intriguing questions regarding how splicing factors, including snRNPs, accommodate the diversity of intronic *cis*-elements in a genome and across diverse genomes. While splicing factors and spliceosome assembly have been extensively studied in intron-poor yeast models, such as *Saccharomyces cerevisiae* and, to a lesser extent, in *Schizosaccharomyces pombe*, their role in intron-rich transcriptome of *Cryptococcus neoformans* where over 99% of transcripts bear multiple introns, remains relatively understudied. Notably, the exon-intron architecture of *C. neoformans*, with degenerate splice signals and variable intronic features, differs from well-studied fungal models and resembles the intronic features found in higher eukaryotes (e.g. plants and humans). Therefore, *C. neoformans* is a promising model to investigate splicing regulation in complex transcriptomes [16].

Here, we studied the genome-wide splicing function of *C. neoformans* Prp16 by generating a promoter-shutdown conditional knockdown strain. We uncovered a genome-wide but intron-substrate-specific role for Prp16 in splicing *C. neoformans* introns. The intron-specific functions are partly contributed by pre-mRNA and U5 loop 1 interactions, which could be related to its role in remodelling the spliceosome for catalysis. Additionally, we report that the splicing of short and ultra-short introns is more sensitive to Prp16 depletion than that of longer introns, suggesting that Prp16 plays a crucial role in processing these specific types of introns.

## Results

### C. neoformans Prp16 is essential for growth and viability

**Figure 1.**
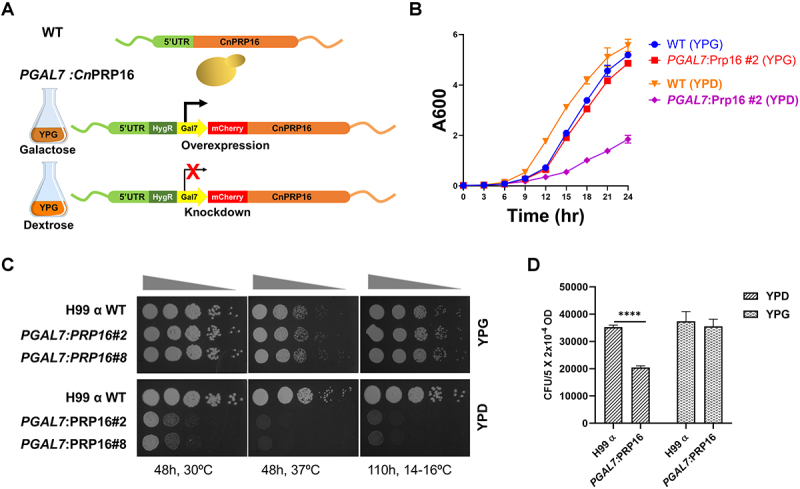
*C. neoformans* Prp16 is essential for growth and viability. (A) Schematic representation of the conditional knockdown *C. neoformans* H99 strain with conditional promoter-shutdown *PGAL7*:mCherry-Prp16 cassette. (B) and (C) the growth kinetics of *C. neoformans* WT and *PGAL7*: Prp16 strains (two independent integrants #2 and #8 with identical growth) in YPG and YPD media. (b) Growth curve at 30°C as recorded by OD_600_ in liquid culture (error bars represent standard deviation of 3 biological replicates). (C) Growth profile by 10-fold serial dilution on agar plates incubated at 30°C, 37°C and 16ºC. (D) Viability of WT and *PGAL7*: Prp16 strains grown in YPG and YPD media for 9 hours and then platted upon YPG plates. Colony forming units (CFUs, shown in y-axis) were calculated from three independent replicate experiments. One-way ANOVA with Tukey’s multiple comparison test was used to calculate significance (ns, *p* ≥ 0.05; * - *p* < 0.05; ** - *p* < 0.01; *** - *p* < 0.001; **** - *p* < 0.001).

*S. cerevisiae* Prp16 is an essential spliceosomal DEAH box helicase that carries out remodelling from the spliceosomal branching conformation formed after the first-step catalysis (C complex) to the second-step exon ligation conformation (C* complex), as deciphered from extensive *in vitro* splicing assays using model actin mini-transcripts. We identified the Prp16 ortholog in *C. neoformans* as CNAG_02303 using a homology-based iterative HMMER [17] jackhammer search, with *S. pombe* Prp16 as the input sequence. The possibility of CNAG_02303 being any other spliceosomal helicase was excluded through comprehensive analysis. We compared proteins closely related to CNAG_02303 in the reference proteomes of *S. cerevisiae, S. pombe, A. thaliana* and Humans. The guide tree derived from multiple sequence alignment demonstrates that CNAG_02303 clusters with Prp16 orthologues from *S. cerevisiae*, *S. pombe*, *A. thaliana*, and *H. sapiens*, and this clade is distinct from the cluster of Prp22 and Prp43 helicase orthologues from these species (Supplementary Figure S1(a)). The identified *C. neoformans* Prp16 ortholog shares significant conservation at the C-terminal helicase domain with all six signatory motifs observed in other DEAD/H box helicases (Supplementary Figure S1(b)). These motifs are involved in ATP-binding, hydrolysis, and RNA duplex unwinding [13,18]. To explore the splicing functions of *C. neoformans* Prp16, we engineered a *C. neoformans* Prp16 conditional knockdown strain by swapping the native promoter with *Gal7* promoter and N-terminal mCherry tag (Figure 1(A)). The resulting strain, *PGAL7*:Prp16, overexpresses the mCherry-Prp16 transcript when grown in permissive galactose media (YPG), and promoter shutdown occurs when grown in nonpermissive glucose (YPD) media (Figure 1(A), Supplementary Figure S2(a, e)). We tested Prp16 transcript levels and mCherry-Prp16 protein levels when grown in YPD media using RT-PCR, confocal microscopy, and western blotting (Supplementary Figure S2(b), S2(c), S2(d)). As *PGAL7*:Prp16 cells were revived and maintained in permissive YPG media conditions where the overexpressed Prp16 protein would accumulate, these cells continued normal growth for the first 12 hours upon shift/transfer to nonpermissive YPD media (Supplementary Figure S2(e)). We therefore standardized growth conditions to achieve significant knockdown of Prp16 by growing primary culture in permissive YPG media followed by growth in non-permissive YPD media for 12 h, followed by re-inoculation again this time into two cultures, one in non-permissive media YPD and another in permissive media YPG conditions for 3–24 h (Supplementary Figure S3(a)). In comparison with similarly treated wild-type (WT) cells, the growth kinetics of *PGAL7*:Prp16 strain was noticeably reduced after 9 h of growth in non-permissive YPD media. No significant growth alteration was observed between *PGAL7*:Prp16 and WT strains when grown in permissive YPG media (Figure 1(B,C)). A similar growth profile was observed by 10-fold serial dilution of cultures spotted on agar plates with permissive and non-permissive media, followed by growth at 30°C, 37°C and 16°C (Figure 1(C)). Notably, the growth of the *PGAL7*:Prp16 strain in non-permissive YPD media is poorer at the low temperature of 16°C as compared to the optimal 30°C. The viability of *PGAL7*:Prp16 strain was significantly reduced compared to that of the WT strain only when grown in YPD media and not in permissive YPG media (Figure 1(D)).

### Conserved residues in the helicase domains of Prp16 are essential for efficient splicing of cellular introns

**Figure 2.**
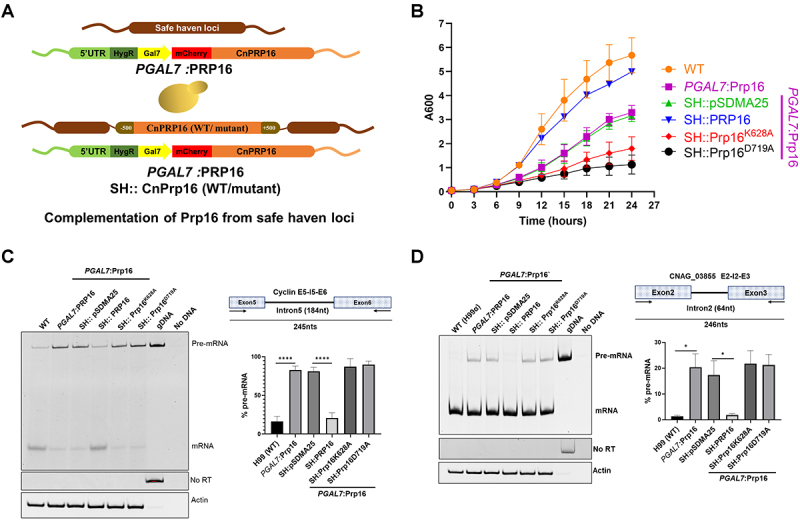
The conserved helicase domain of Prp16 is essential for splicing. (A) Schematic representation of strain expressing full-length CnPRP16 or mutant proteins from the safe haven locus. (B) Growth kinetics of *C. neoformans* WT, *PGAL7*:Prp16 and *PGAL7*:Prp16 strains expressing WT CnPRP16 or its mutants K628A and D719A from safe haven locus. All the strains were grown first in permissive YPG media then subcultured in YPD media for 12 hours. Culture aliquots (at 0.05 OD) were inoculated in YPD media growth measured by recording OD_600_ at 3 hours intervals. The data was plotted with error bars representing standard deviation of 3 biological replicates. (C) and (D) Splicing assays for cellular introns (C) CLN1 Intron5 and (D) CNAG_03855 intron2, by semi-quantitative RT-PCR on RNA isolated from *C. neoformans* WT, *PGAL7*:Prp16 and *PGAL7*:Prp16 strains expressing WT CnPRP16 or the mutants K628A or D719A from safe haven locus. All strains were grown in conditions standardised for Prp16 knockdown (YPD), as described previously. Quantification was done from three biological replicates. One-way ANOVA with Tukey’s multiple comparison test was used to calculate significance (ns, *p* ≥ 0.05; * - *p* < 0.05; ** - *p* < 0.01; *** - *p* < 0.001; **** - *p* < 0.001).

Biochemical assays with *S. cerevisiae* Prp16 ascribed its catalytic activity to ATP hydrolysis and conformational remodelling of the spliceosome from the C complex to the C* complex by the unwinding of RNA duplexes [18,19]. Conserved motifs in the Prp16 C-terminal helicase domain are essential for ATP binding, hydrolysis, and RNA duplex unwinding. *S. cerevisiae* Prp16 mutants in the conserved motifs cause a dominant negative effect on growth [19]. To gain functional insights into *C. neoformans* Prp16, we generated alanine substitution mutants in the conserved helicase motif 1 (GSGKT) residue K628A and motif 2 (DEAH) residue D719A (Supplementary Figure S3(b)). Homologous mutations in *S. cerevisiae* Prp16, K379A and D473A, fail to catalyse the second step of splicing in yeast actin mini-transcripts and competitively inhibited splicing when supplemented into splicing reactions initiated with wild-type yeast extract [18]. In the *PGAL7*:Prp16 strain, we expressed from a heterologous safe haven locus the CnPrp16 wild type, K628A or D719A allele, where their expression was driven by the endogenous Prp16 promoter (Figure 2(A)). These strains with ectopic expression of wild-type or mutant CnPrp16 alleles exacerbated the growth defect in *PGAL7*:Prp16 when grown in YPD medium (Figure 2(B) and Supplementary Figure S3(c)). The splicing efficiency of selected introns with diverse lengths and intronic *cis* consensus elements was tested. Cln1 intron 5, CNAG_03855 intron 2, and Cas35 intron 7 were chosen to assess splicing efficiency (Figure 2(C,D) and Supplementary Figure S3(d)). For these introns, accumulation of unprocessed pre-mRNA and a concomitant decrease in spliced mRNA were observed in the *PGAL7*:Prp16 strain grown in YPD medium (Figure 2(C,D) and Supplementary Figure S3(e)). Consistent with the rescue of growth defects when wild-type Prp16 was expressed ectopically from the safe haven locus, we also noted a rescue of the splicing defect of these cellular introns in the *PGAL7*:Prp16 SH:PRP16 strain. The splicing profile of these introns remained compromised in *PGAL7*:Prp16 SH:Prp16^K628A^ and *PGAL7*:Prp16 SH:Prp16^D719A^ strains (Figure 2(C,D) and Supplementary Figure S3(e)). These data, together with prior literature on *S. cerevisiae* Prp16, suggest that the splicing defects observed in *PGAL7*:Prp16 are dependent on the catalytic activity of CnPrp16.

### Prp16 knockdown in *C. neoformans* alters genome-wide splicing profile

**Figure 3.**
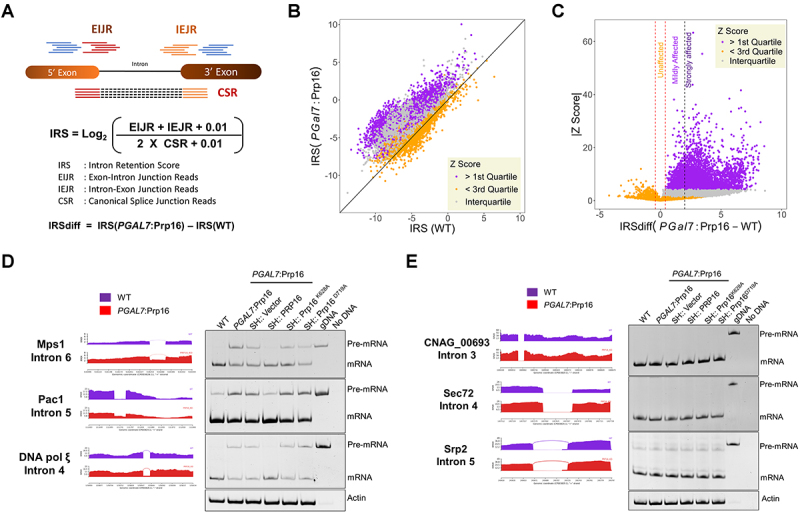
Genome-wide splicing profile on *C. neoformans* Prp16 knockdown. (A) Schematic representation of the algorithm used for genome-wide splicing analysis. (B) Scatter plot of intron retention score (IRS) in *PGAL7*:Prp16 versus WT cells. Each data point represents each annotated intron with a sufficient number of junction reads. The introns are classified based on quartiles of the Z-score of each intron. (C) Plot of absolute Z score against IRSdiff (difference of IRS between *PGAL7*:Prp16 and WT). The vertical red dotted lines represent the cut-off applied to classify unaffected and affected introns, and the vertical dotted black line further demarcates mildly affected and strongly affected introns. (D) Validation of three affected introns with large IRS differences in the RNAseq data and by independent semi-quantitative RT-PCR assays. Splicing defect in the *PGAL7*:Prp16 strain was rescued by ectopic expression of wild type CnPRP16 (SH:PRP16), but not in strains that express helicase domain mutants (SH:Prp16^K628A/D719A^). (E) Validation by semi-quantitative RT-PCR of the splicing status of three unaffected introns in RNAseq data. No pre-mRNA accumulation was seen in the *PGAL7*:Prp16. No pre-mRNA accumulation in strains with ectopic expression of helicase domain mutants of Prp16 (SH:Prp16^K628A/D719A^). For RT-PCR validation, all the strains were grown in conditions standardized for Prp16 knockdown (YPD), as described previously.

Next, we assessed genome-wide splicing consequences of the knockdown of Prp16 in *C. neoformans*. Deep next-generation sequencing of cellular RNAs isolated from three biological replicates of the WT and *PGAL7*:Prp16 strains grown in YPD media was performed. We achieved high quality and high depth of reads from all samples, as reflected by 60–80 million total read counts, of which more than 95% were aligned to the *C. neoformans* genome. As expected, due to promoter shutdown, a decrease in reads aligned to the Prp16 locus was observed for *PGAL7*:Prp16 cells compared to WT (Supplementary Figure S4(a)). Genome-wide splicing analysis was performed by adopting an algorithm previously used for the analysis of the fission yeast *S. pombe* transcriptome [20]. Based on *C. neoformans* genome annotation (GTF) this algorithm uses the number of mapped reads traversing exon-exon junctions (EEJR), exon-intron junctions (EIJR) and intron-exon junctions (IEJR) of each intron to calculate the intron retention score (IRS) for each intron. This IRS score is a quantitative representation of splicing efficiency (Figure 3(A)). Introns with a total number of junction reads of < 10 were filtered out from the analysis.

An overall shift in the mean IRS values in *PGAL7*:Prp16 cells grown in YPD indicated the genome-wide role of Prp16 in the splicing of the majority of introns (Figure 3(B) and Supplementary Table S3). This reiterates the near-universal role of Prp16 in splicing in *C. neoformans*. As IRS values exhibited a normal distribution (Supplementary Figure S4(b) and S4(c)), two-sample Z tests were performed with a null hypothesis of equal IRS between WT and *PGAL7*:Prp16 to calculate the significance of altered splicing, and a Z score was assigned to each intron. The values for all introns were sorted by descending intron Z-scores and divided into quartiles. Introns with Z-score values in the > 1st quartile exhibited significant changes in splicing efficiency upon Prp16 knockdown, and introns with Z-score values in the < 3rd quartile exhibited the least significant changes in splicing upon Prp16 knockdown. This data aligns with the scatter plot of IRS (Figure 3(B) and Supplementary Table S3), where introns in the > 1st quartile show a greater bias towards Prp16 knockdown (purple data points) and introns in the < 3rd quartile lie along the diagonal line (orange data points). The IRS values that fall within the interquartile Z score (falling between the 1st and 3rd quartiles) overlap with the IRS distribution of introns in the > 1st quartile. This may have arisen from the variations in IRS values observed across different RNA replicates for some affected introns, which resulted in a lower (interquartile) Z score. For the categorization of affected and unaffected introns, the difference in IRS values between *PGAL7*:Prp16 and WT (IRSdiff) was plotted against the absolute Z score (|Z score|) (Figure 3(C)). Based on this plot, the minimum IRSdiff value obtained for the > 1st quartile was assigned as the cut-off between the affected and unaffected introns (Figure 3(C), vertical red dotted lines). Affected introns were further divided into strongly and mildly affected introns based on whether the IRSdiff value was greater than or less than 2. This analysis revealed that > 88% of introns (25,642) were affected by the knockdown of Prp16, and < 12% (2808) remained unaffected upon Prp16 knockdown (Supplementary Figure S4(d)). A small set of 685 introns had negative IRSdiff values and negative Z-scores. We surmise that this group could be false positives from the unaffected category of introns, but where transcripts had either an increase in the mRNA read count or a decrease in the pre-mRNA read count. This set of 685 introns was not used for further analyses.

The data from the bioinformatics analysis were validated by semi-quantitative RT-PCR for some introns chosen from the strongly affected and unaffected categories (Figure 3(D,E)). Consistent with the transcriptomic data, Mps1I6 (Mps1 Intron6), Pac1I5 (Pac1 Intron5), and DNApolξ I4 (DNApolξ Intron 4) from the strongly affected category showed RT-PCR amplicons, denoting increased pre-mRNA accumulation in *PGAL7*:Prp16 cells grown in YPD media. Expression of wild-type Prp16 from the heterologous safe haven locus rescued the impaired splicing of all three introns (Figure 3(D), lane 4). Ectopic expression of helicase DEAD box mutant alleles Prp16 K628A and D719A from the safe haven locus (*PGAL7*:Prp16 SH:Prp16^K628A^ strain and *PGAL7*:Prp16 SH:Prp16^D719A^) could not rescue the splicing defect triggered by the knockdown of Prp16, as evident from the high levels of unspliced precursor (Figure 3(D), lanes 5 and 6). For the introns CNAG_00693I3, Srp2I5, and Sec72I4 from the unaffected category, the RT-PCR assay also showed that the splicing profile was unaltered in *PGAL7*:Prp16 cells grown in repressive media (Figure 3(E)). Interestingly, splicing of these introns remained unaffected even after ectopic expression of helicase DEAD box mutant alleles from the safe haven locus in *PGAL7*:Prp16 cells. This suggests that splicing of these introns could be Prp16-independent splicing, as the predicted dominant negative effect of the K628A and D719A mutants was not observed.

### Exonic consensus elements at pre-mRNA 5’splice site and 3’ splice site corelate with Prp16 dependent efficient splicing

**Figure 4.**
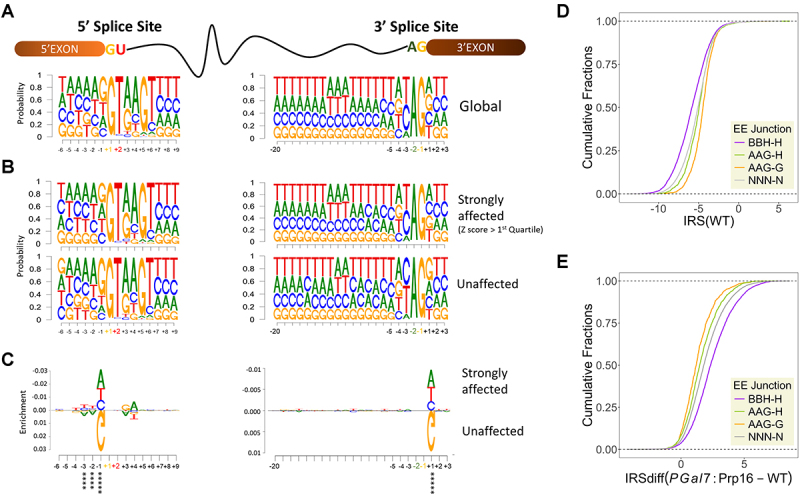
Intronic features determining intron dependency on Prp16. (A) Sequence logo for 5’splice site and 3’ splice site for total annotated *C. neoformans* introns. (B) Sequence logo for 5’splice site and 3’ splice site for strongly affected introns with Z score > 1st quartile (upper panels) and unaffected introns (lower panels). (C) DiffLogoLogo analysis was comparing differential sequence enrichment of the 5^′^ SS and 3’SS between strongly affected introns high Z score (>1st quartile) and unaffected introns. The enrichment of A, A and G against other nucleotides at positions − 3, −2, and − 1 of 5’SS of unaffected introns was significant by p-values under 6.9 × 10^−12^, 7.2 × 10^−14^ and 3.9 × 10^−105^ by two-sided Fisher’s exact test, respectively. The enrichment of G against other nucleotides at positions + 1 of 3’SS of unaffected introns was significant by p-values under 1.5 × 10^−27^ by two-sided Fisher’s exact test. (D) Cumulative plot of IRS in WT for each group of classified introns. All introns are classified into four groups based on their last three nucleotides of 5’exon and 1^st^ nucleotide of 3’exon. (E) Cumulative plot of IRS differences for each group of classified introns in *PGAL7*:Prp16 versus WT.

Next, we assessed the *cis* features in introns and at exon-intron junctions for introns where splicing was strongly compromised upon Prp16 knockdown as compared to introns where splicing was unaffected. To this end, we characterized the 5’ splice site (5’SS) and 3’ splice site (3’SS) of all ~ 40,000 *C. neoformans* introns, 4954 introns strongly affected introns (Z score > 1st quartile) in *PGal7*:Prp16 cells, and the set of 2808 unaffected introns (Figure 3(C) Supplementary Figure S4(d)). As per *C. neoformans* genome annotation, sequence logos were generated for 5’SS and 3’SS of all three intron groups (Figure 4(A,B)). Differential enrichment or depletion of specific nucleotides around the 5’SS and 3’SS of strongly affected and unaffected introns was visualized using DiffLogo (Figure 4(C)). Notably, the last 3 nucleotides preceding the 5’SS are enriched for AAG in the set of unaffected introns and the 1st nucleotide of 3’exon, that is, downstream of 3’SS more frequently a G in the unaffected introns (Figure 4(C)). Further, we deduced that intronic N3 and N4 nucleotides at the 5’SS being “GA” is also a signature common to affected introns (Figure 4(C)). No differential enrichment was observed between the affected and unaffected introns for intronic sequences at the poly-pyrimidine track and the 3’SS preceding the 3’ exon (Figure 4(C)).

*In vitro* splicing assays for Prp16 activity using human or *S. cerevisiae* splicing extracts showed its role in remodelling splicing complexes that allow the 5’ and 3’ exons in a conformational alignment that promotes the second step of catalysis. This is facilitated by U5-snRNA loop1 interactions with the 5’ and 3’ exons [21]. Therefore, we focused our next analysis on *cis* sequences located at the end of 5’exons and at the start of 3’ exons, which were found to be differentially enriched in unaffected introns as compared to strongly affected introns (Figure 4(C)). Introns were classified into four groups based on the last three nucleotides of the 5’exon and the first nucleotide of the 3’ exon: AAG-G (872 introns), AAG-H (1896 introns), BBH-H (5596 introns), and NNN-N (20671 introns). Next, we assessed the splicing efficiency of these intron classes by calculating the IRS in the WT and plotting the cumulative distribution plots. Notably, in the WT strain, introns with the BBH-H signature were the most efficiently spliced, whereas those with the AAG-G signature were the least efficiently spliced (Figure 4(D) and Supplementary Figure S5 (a)). The splicing defect upon Prp16 knockdown was assessed for these classes by calculating the IRSdiff and plotting cumulative distribution plots. Consistent with the consensus sequence seen by the SeqLogo and DiffLogo algorithms, we see that introns with AAG-G signature are the least affected and the BBH-H signature is the most affected by the depletion of Prp16 (Figure 4(E) and Supplementary Figure S5(b)). This suggests that Prp16 plays a role in enhancing the efficiency of splicing of introns with sequence diversity in the last three nucleotides of the 5’ exon that precede the 5’SS and the first nucleotide of the 3’ exon that follows the 3’SS.

### Pre-mRNA substrates with weaker interactions with U5 snRNA loop1 are Prp16 dependent

**Figure 5.**
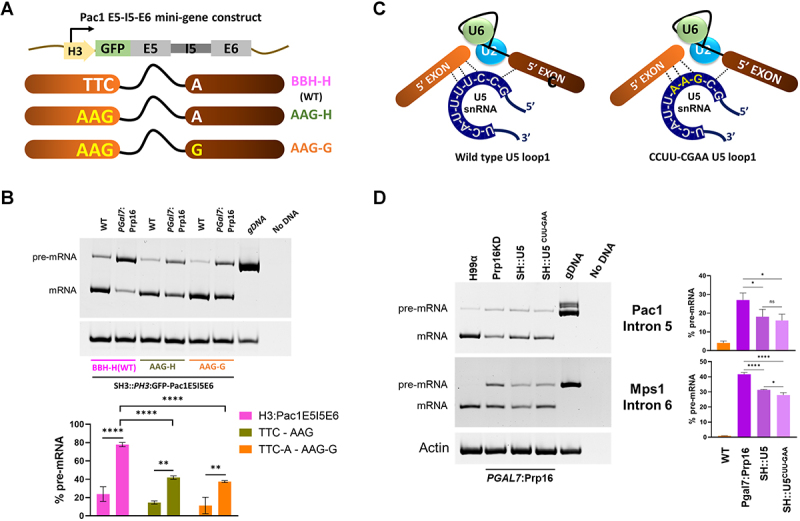
Interactions between U5 loop1 and pre-mRNA contribute to Prp16 dependency (A) schematic representing the Pac1E5I5E6 minigene construct and mutants generated at 5’ exon and 3’exon. (B) Semi-quantitative RT-PCR for Pac1E5I5E6 minigene expressed from safe haven 3 locus in WT and *PGAL7*:Prp16 strains. (C) Schematics for U5 pre-mRNA interactions before exon ligation for wild type U5 and loop1 mutant U5^CUU-GAA^. (D) Semi-quantitative RT-PCR of cellular Pac1E5I5E6, Mps1E6I6E7 in WT, *PGAL7*:Prp16 and *PGAL7*:Prp16 strains ectopically expressing wild type U5 and U5^CUU-GAA^ from safe haven locus. Semi-quantitative RT-PCR was quantified from three biological replicates. One-way ANOVA with Tukey’s multiple comparison test was used to calculate significance (ns, *p* ≥ 0.05; * - *p* < 0.05; ** - *p* < 0.01; *** - *p* < 0.001; **** - *p* < 0.001).

To experimentally validate the interpretations emerging from the bioinformatics analysis of splice sites in Prp16 dependent and independent introns, we designed minigenes to be tested for their splicing efficiency in *C. neoformans* cells. We chose Pac1 intron5 and its flanking exons (E5I5E6) from the set of strongly affected introns in the Prp16 knockdown dataset. The construct containing this minigene under the control of the H3 promoter was cloned in an integration vector pEE27 with flanking sequences suitable for integration into the heterologous safe haven 3 locus [22]. Thus, once integrated into the WT or *PGal7*:Prp16 strains, mini-transcript expression and its splicing status can be assessed. We found that the mini-transcript splicing efficiency was similar to that observed for intron 5 in the endogenous Pac1 transcript (Supplementary Figure S5 (c) and S5(d)). We generated and tested two variants using these splicing mini-transcript assays. The first where the last three nucleotides of 5’ exon in the mini-transcript were mutated from UUC to AAG and the second where these 5’exon mutations were combined with A to G change in the first nucleotide of 3’ exon of the mini-transcript (Figure 5(A)). These variant mini-transcripts were also expressed from the safe haven 3 locus in the WT and *PGal7*:Prp16 cells. The strong splicing defect of the mini-transcript in Prp16 depleted cells was alleviated when the 5’SS exonic UUC sequence was mutated to AAG, as the mini-transcript intron was spliced more efficiently in *PGal7*:Prp16 cells grown in YPD (Figure 5(B), lane 2 *vs*. lane 4, bar graphs pink *vs*. green). For mini-transcripts with A to G mutation in the 1st nucleotide of 3’exon that also had 5’SS exonic UUC to AAG nucleotide mutations (Figure 5(B), lane 6), the splicing efficiency was marginally improved as compared to mini-transcript with only 5’exonic mutations (compare *PGal7*:Prp16 lanes 4 *vs*. 6 Bar graphs green *vs*. orange). However, this improved splicing efficiency of the AAG-G mutant Pac1 I5 mini-transcript under conditions of Prp16 depletion did not approach the efficiency of splicing observed for other endogenous cellular transcripts with the AAG-G *cis* signature, which are spliced well even after Prp16 depletion. This suggests that additional factors apart from exonic sequences at 5’SS and at 3’SS could contribute to the requirement of Prp16 for efficient splicing.

As an orthogonal approach, we examined whether U5 snRNA interactions with pre-mRNA *cis-*elements could influence the splicing status of introns that are not spliced well under Prp16 knockdown conditions. The U5 locus, along with the 500 bp upstream genomic region (as an endogenous promoter) and 500 bp downstream genomic region, was cloned into the integration vector pSDMA57, which is specific for integration into the safe haven 1 locus [23]. We also created mutants in U5 loop 1 residues in the pSDMA57 clone that likely improve the base-pairing interaction with exonic sequences in the cellular transcript for Pac1E5I5E6 pre-mRNA (Supplementary Figure S6 (b)). To achieve this, we mutated the cloned U5 sequence in pSDMA57 to express mutant U5 with the loop1 sequence as a GAA (Figure 5(C)). The U5^WT^ and U5^CUU-GAA^ mutant expression constructs in the pSDMA57 vector were integrated at the safe haven 1 locus of *PGAL7*:Prp16 strains (Supplementary Figure S6(a)). In RT-PCR assays, we observed that the expression of this additional copy of wild-type U5 and U5^CUU-GAA^ in *PGAL7*:Prp16 strains improved the splicing efficiency of cellular Pac1 E5I5IE6. This improved splicing was manifested by increased mRNA, but no change in pre-mRNA was observed (Figure 5(D) lane 2 *vs*. lane 3). This may indicate a rescue/improved efficiency of the second splicing step for Pac1E5I5E6. Consistently, in *PGAL7*:Prp16 cells expressing the U5^CUU-GAA^ mutant, we observed slightly better splicing of cellular Pac1 E5I5IE6 as compared to *PGAL7*:Prp16 cells that express wild-type U5 from the safe haven locus (Figure 5(D), top panel lane 2 *vs*. lane 4). We also examined the splicing of Mps1 E6I6E7, where pre-mRNA interactions with U5 loop 1 were not canonical Watson Crick base-pairing interactions. Splicing efficiency was assessed in cells expressing either U5 or U5^CUU-GAA^ from the safe haven locus (Supplementary Figure S6(b)). As seen for Pac1 I5 splicing, we noted improved splicing of Mps1 E6I6E7 upon expression of an additional copy of wild-type U5 in *PGAL7*:Prp16 cells (Figure 5(D), lower panel lane 2 *vs*. 3). Furthermore, in *PGAL7*:Prp16 SH:U5^CUU-GAA^ cells where U5 loop1 interacts with Mps1 E6I6E7 are altered, we noted a marginally better splicing of Mps1 intron 6 as compared to its splicing in *PGAL7*:Prp16 SH:U5 cells (Figure 5(D), lane 3 *vs*. 4). It is plausible that U5^CUU-GAA^ transcript levels, as expressed by U5 integrated at safe haven locus are inadequate to efficiently compete with endogenous U5 and consequently have only subtle changes in the splicing status of cellular transcripts. These results support the hypothesis that stronger pre-mRNA recognition by increased expression of wild-type U5 snRNA or even the U5^CUU-GAA^ mutant may suppress intron retention caused by Prp16 depletion.

### Prp16 promotes the splicing of short and ultra-short introns in *C. neoformans* genome

**Figure 6.**
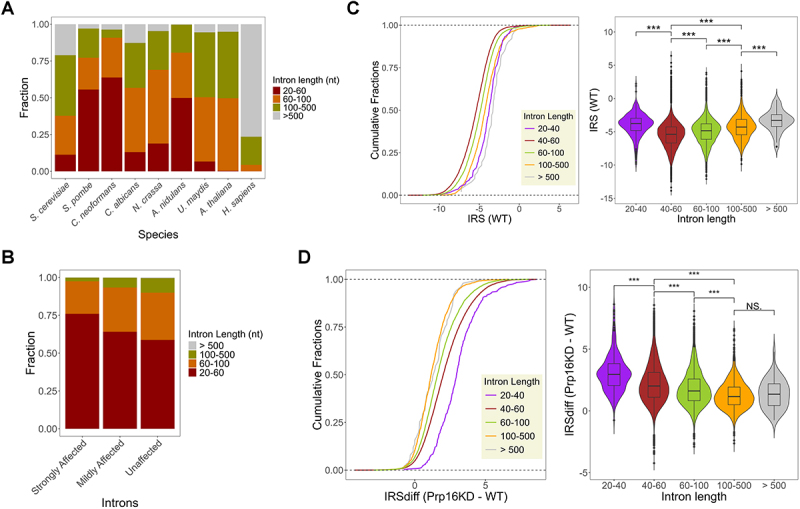
(A) Stacked bar plot for intron sizes across different species. *C. neoformans* possess a maximum fraction of short and ultra-short introns among all species. (B) Stacked bar plot for the occurrence of various size introns in the transcriptome of Prp16 knockdown classified as strongly affected, mildly affected and unaffected. (C) Cumulative plot of intron retention score (IRS) for each group of classified introns in WT transcriptome. All introns are classified into five groups based on their length: 20–40 nts, 40–60 nts, 60–100 nts, 100–500 nts and > 500 nts. The right panel shows a box plot of IRS in WT for each group of introns. (D) Cumulative plot of IRS difference between *PGAL7*:Prp16 and WT for each group of classified introns. The right panel shows a box plot of IRS differences between *PGAL7*:Prp16 and WT for each group of introns. Asterisks indicate statistically significant differences, as determined by the Wilcoxon rank-sum test within the R package ggplot2.

The EM structure of human spliceosomal complex A formed after the assembly of U1 and U2 snRNPs covers approximately 79–125 nts of RNA substrate [24]. The remarkable variability of intron lengths from < 50 nts to > 50,000 nts [25] seen in several genomes poses questions regarding the assembly of the spliceosomal complex on short and ultra-short introns without steric hindrance. Comparative analysis of intron length distribution across diverse species revealed a higher prevalence of short and ultra-short introns in most fungal genomes studied thus far. Strikingly, *C. neoformans* harbours the highest percentage of short (60–100 nts) and ultra-short (<60 nts) introns among all the studied fungal species (Figure 6(A)). In the dataset of introns affected by CnPrp16 depletion, we observed a higher representation of short introns in the strongly and mildly affected intron categories than in the unaffected group of introns (Figure 6(B)). We analysed the splicing efficiency of introns after categorizing them based on intron length, using finer bin sizes for the ultra-short intron group. The CDF plot of IRS values in the WT dataset (Figure 6(C)) suggests *C. neoformans* ultra-short introns 40-60nt (dark red line) are spliced most efficiently, followed by short introns 60–100nt (light green line), introns in the range 100–500 nts (orange line), and the poorest efficiency even in wild-type cells is for long introns > 500nt (grey line). A small population of introns shorter than 40nt (purple lines) showed the highest IRS value, suggesting that this group had notably poor splicing efficiency. These data open an intriguing interpretation that *C. neoformans* spliceosomal machinery is apparently geared for recognizing and splicing a majority of its genome’s ultra-short and short introns (covering lengths of 40-100nts). Next, we analysed splicing defects in introns binned into groups based on their length under Prp16 knockdown conditions. Here, we calculated and plotted the IRSdiff value for introns categorized based on size (Figure 6(D)), and observed a higher splicing defect (IRSdiff) for ultrashort introns (20–40 nts and 40–60 nts, purple and dark red lines) followed by short introns (60–100 nts, light green line). This correlation between splicing defects upon depletion of Prp16 (IRSdiff) and intron length is significant when examined even when scanned at 10 nts resolution (Supplementary Figure S6(c) and S6(d)). This correlation was seen only for introns from 20nt to 80nt introns as no significant differences in IRSdiff scores were observed for introns classes 80–90 nts, 90–100 nts and > 100 nts (Figure 6(D) right panel and Supplementary Figure S6(d)). Thus, the role of Prp16 in the efficient splicing of ultrashort and short introns in *C. neoformans* transcriptome was deduced.

## Discussion

The dynamic assembly, activation, and catalysis of the spliceosome involve several remodelling steps driven by eight helicases, including DEAD and DEAH-box helicase proteins [4]. Prp16, a DEAH-box helicase, was detected in step one spliceosomes by cryo-EM of human and yeast complexes [26,27]. Single-molecule and cross-linking experiments suggested that Prp16 binds to the single-stranded RNA region downstream of the branchpoint and translocates on the lariat intermediate as a molecular winch [28,29]. The ATPase activity of Prp16 facilitates the conversion of the branching C complex to the step-two catalytically activated C* complex [30]. The roles of splicing factors in development and disease have been reported in several models [31–33]. Interestingly, the autosomal recessive mutation G332D in DHX38, the human ortholog of Prp16, is associated with retinitis pigmentosa [34]; however, its requirement for introns with diverse sizes and splicing signatures remains understudied.

In this study, we report genome-wide splicing defects in the short intron-rich genome of *C. neoformans* triggered by Prp16 knockdown. We found that a small subset of introns with flanking exonic sequences that can be robustly recognized by U5 snRNAs were spliced well under Prp16 knockdown conditions. While complementation of splicing defects in knockdown conditions is achieved by the expression of wild-type Prp16 from a heterologous locus, *C. neoformans* strains expressing Prp16 mutants in catalytic motif I (K628A) and motif II (D719A) grow poorly and remain splicing defective. Interestingly, these phenotypes are similar to observations on the *S. cerevisiae* Prp16 mutants K379A and D473A in homologous residues, which are synthetic lethal in the *S. cerevisiae prp16Δ* null mutant and cause dominant negative growth impairment when overexpressed in wild-type yeast [19]. *In vitro*, these *S. cerevisiae* Prp16 mutant proteins are unable to catalyse the second step of splicing in budding yeast splicing extracts depleted of Prp16, and when supplemented to reactions with wild-type extracts, these mutant proteins inhibit splicing [18]. Consistent with these observations, the K628A and D719A alleles of *C. neoformans* Prp16 exacerbated the growth defect of *PGAL7*:Prp16 strains grown in non-permissive media, indicating dominant negative growth suppression, as observed in *S. cerevisiae*. Furthermore, the K628A and D719A alleles of *C. neoformans* Prp16 do not alter the splicing defects triggered by Prp16 knockdown cells, suggesting that the splicing defects caused by Prp16 depletion are predominantly associated with the catalytic activity of *C. neoformans* Prp16.

**Figure 7.**
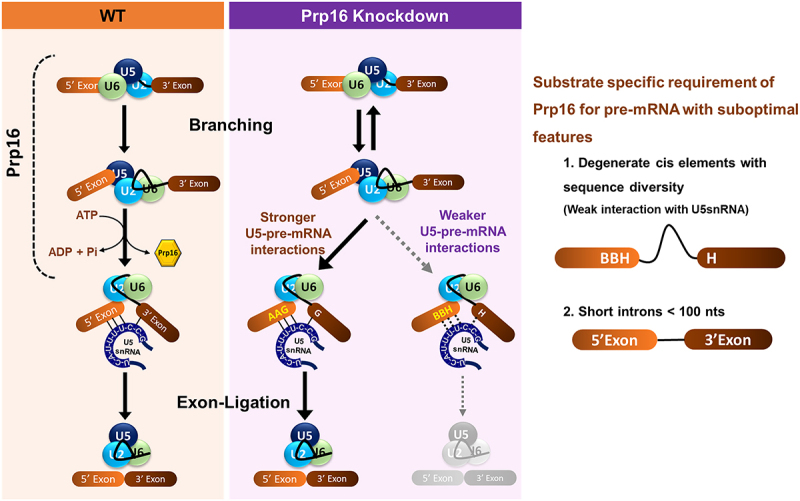
Schematic illustration of the two spliceosomal catalytic reactions in WT and Prp16 knockdown condition. In WT, ATP-dependent helicase activity of Prp16 catalyses conformational transition from branching conformation after first step catalysis to exon ligation conformation primed for the second step. In Prp16 knockdown conditions (*PGAL7*:Prp16), these conformational transitions are efficient for introns with stronger U5 loop1-pre-mRNA interactions (AAG-G). However, introns with weaker U5 loop1-pre-mRNA interactions (BBH-H) require Prp16 to remodel the branching conformation to exon ligation conformation. The right panel depicts that Prp16 enables the splicing of introns with diverse exonic sequences at 5’SS and 3’SS. Additionally, Prp16 also requires the splicing of short and ultra-short introns.

Genome-wide transcriptome analysis revealed that Prp16 is required for splicing of the vast majority of *C. neoformans* introns. Interestingly, a small subset of introns is spliced efficiently even after Prp16 depletion, and these introns are also spliced in strains expressing Prp16 K628A and D719A mutants. A significant proportion of these introns spliced independent of Prp16 are enriched for AAG residues as the last three nucleotides of their 5’ exon (−3, −2 and −1 positions of 5’SS) and with G residue as 1st nucleotide of their 3’ exon (+1 position following the 3’SS). In *S. pombe* and *A. thaliana*, introns where the 5’exons end with AAG are strongly recognized by U5 snRNA and can suppress the splicing defect triggered by mutations in the U6 m6A methyltransferase [20,35]. Statistical analysis of the human transcriptome suggests that the G nucleotide at the end of the 5“ exon and at the start of the 3” exon favours a strong binding register for U5 loop1 C39|C38 and significantly contributes to specific exon recognition and splicing precision [21]. Our analysis revealed that the U5 snRNA-pre-mRNA intron interaction in *C. neoformans* transcripts is one factor determining the requirement of Prp16 for efficient splicing of each intron. In budding yeast *in vitro* splicing assays, the U5.U6/U4 tri-snRNP assembled in the spliceosome allows U5 loop 1 recognition of these three terminal nucleotides of the pre-mRNA 5^′^ exon. After the first step of catalysis, this interaction ensures that the 5’ exon is tethered to the spliceosome. This interaction also aligns and allows U5 to interact with the 3’ exon in the C* complex for second-step catalysis [36,37]. Our global transcriptome study inferred that pre-mRNA exonic AAG-G sequences that strongly interact with U5 loop 1 could favour conformational transitions even when *C. neoformans* cells were depleted for Prp16 activity. In contrast, introns with weaker pre-mRNA exonic interactions with U5 loop1 (*e.g.*, BBH-H) require Prp16-driven remodelling for spliceosomal transitions (Figure 7). This inference is consistent with other data from our mutational analysis of mini-transcripts, where “BBH-H” exonic signatures were mutated to “AAG-G” in a model minigene and were tested in cells depleted of Prp16. The significantly enhanced splicing efficiency of the mutant mini-transcript supports the interpretation that strong U5 loop 1 interactions with the 5’ exon could strengthen the anchoring of the 5’SS for spliceosome activation. Since we note the conversion of only 5’ exon ‘BBH’ to ‘AAG’ signatures also enhances the splicing of mini-transcript in Prp16 knockdown conditions this opens the speculation of a role for *C. neoformans* Prp16 prior to first step splicing catalysis. We have also reported that increased U5 snRNA levels can improve the splicing efficiency of Prp16-dependent introns. These data and our studies on the effect of U5 loop 1 mutant support the hypothesis that the interaction of U5 snRNA with pre-mRNA contributes towards the requirement of Prp16 for the efficient splicing of *C. neoformans* transcripts.

A notable feature of the *C. neoformans* is the high prevalence of short introns. In the *C. neoformans* genome, the average intron size is 56 nt [38]. Human spliceosomal complex A with U1 and U2 snRNPs is a ~ 26 × 20 × 19.5 nm globular and asymmetric particle that can cover 9–125 nts of linear RNA upon assembly [24,39]. However, all organisms possess a fraction of introns in their transcriptome that are much shorter than 79 nucleotides. For instance, studies have reported human ultra-short introns with lengths as short as 43–65 nts, which are efficiently spliced [25,40]. The mechanisms underlying for recognizing the *cis*-elements in short introns by the spliceosome without steric hindrance remain unknown. A recent study revealed that the human alternative splicing regulator SPF45 (RBM17) is essential for efficient splicing of many short introns. SPF45 competes with U2AF^65^ for binding to the U2 snRNP protein SF3B1 and facilitates spliceosomal assembly on short introns with short or truncated polypyrimidine tracks [24]. The ultra-short intron-rich genome of *C. neoformans* is equipped with a spliceosomal machinery that is efficient for the recognition and splicing of these ultra-short introns. As we observed in our study, there is an increased proportion of short and ultra-short introns among the group of introns affected by depletion of Prp16, suggesting a role for Prp16 in splicing *C. neoformans* ultra-short introns. In budding yeast, spliceosomal helicases, such as Prp16 and Prp22, bind to the spliceosome transiently and at a distance downstream from the RNA structure that they remodel [28,29]. This feature eliminates the intron size restriction for Prp16-mediated spliceosome remodelling and opens avenues for future studies in *C. neoformans* to understand the mechanism of splicing of ultra-short introns that could rely on *in vivo* approaches on model mini-transcripts.

Taken together, our study uncovered a genome-wide but substrate-specific function of Prp16 in *C. neoformans*. Intronic features associated with the strength of U5 loop 1–pre-mRNA interactions determine the requirement of Prp16 for splicing and are likely relevant for spliceosomal conformational transitions to ensure splicing efficiency. Furthermore, we underscore the role of Prp16 in splicing ultra-short introns prevalent in the *C. neoformans* genome. This work also shows that pre-mRNA substrates possessing optimal intronic features, such as a strong snRNA-binding register and sufficient length, are capable of undergoing splicing, even in a minimal spliceosome. Conversely, suboptimal introns necessitate the involvement of splicing factors, such as Prp16, for stable non-Watson-Crick interactions with spliceosomal snRNAs for efficient splicing.

## Materials and methods

### Yeast strains, primers, and media used in this study

The strains and primers used in this study are listed in the Supplementary Tables S1 and S2, respectively.

### Construction of *C. neoformans* Prp16 promoter shutdown conditional knockdown

To generate promoter shutdown conditional knockdown of Prp16, we replaced the endogenous Prp16 promoter with the GAL7 promoter. The 1KB fragment upstream of the Prp16 start codon was PCR-amplified from the H99 genomic DNA and cloned into the pBSKS+ vector using the oligos listed in S2 table. Similarly, a 1KB fragment from the start codon of Prp16 was amplified by PCR and cloned into the pBSKS+ vector. After validation by sequencing, the two fragments were subcloned into the pGAL7 mCherry vector. The upstream fragment was cloned at the SacI site, and the downstream fragment was cloned at the HindIII-XhoI site to obtain the final clone, where the reading frame of mCherry was in translational fusion with the N-terminal of Prp16. The resulting plasmid was partially digested with SacI, and the other SacI at the 3’ end of the upstream fragment was deleted by end-filling. The resulting construct was digested to release the homologous recombination cassette, which was released by SacI and Xho1 digestion and introduced into wild-type H99 cells by biolistic transformation. Hygromycin-positive transformants were confirmed by locus-specific PCR using the oligos listed in Supplementary Table S2.

### Construction of safe haven expression constructs for complementation or for expression of mini-transcripts

To complement the Prp16 knockdown strain with wild-type Prp16, the CnPRP16 locus was cloned along with 500bp upstream and 500bp downstream intergenic regions in the safe haven integration vector pSDMA25. To generate mutants of Prp16 at specific amino acids in the conserved catalytic motifs, overlap PCR was performed using mutagenic primers specific to the K628A and D719A sites along with the Prp16 primers listed in Supplementary Table S2. The resulting PCR products were cloned into the pSDMA25 vector and validated by sequencing. The pSDMA25 vector containing the wild-type or catalytic mutant allele of Prp16 was linearized with the BaeI restriction enzyme and introduced into the *PGAL7:Prp16* strain by biolistic transformation. NAT-resistant colonies were screened and validated by locus-specific PCR, using the primers listed in Supplementary Table S2. To generate a strain that expresses U5 snRNA from the safe haven locus, the U5 locus along with 500bp upstream and 500bp downstream region, was first cloned in the safe haven integration vector pSDMA57. The residues in loop 1 of U5 were mutated via inverse PCR using mutagenic primers in the pSDMA57 vector.

### Media and growth conditions

Unless mentioned otherwise, for all experiments, strains were initially grown in permissive media YPG (2% peptone, 2% galactose, 1% yeast extract) overnight and then shifted to non-permissive media YPD (2% peptone, 2% glucose, 1% yeast extract) for 12 h. From these 12 hour YPD grown cultures, an aliquot of 0.1O.D. was subcultured into either permissive YPG media (for conditional overexpression of mCherry-Prp16) or non-permissive YPD (for conditional knockdown of mCherry-Prp16) media and cultures were grown for 9 h. All the strains were grown at 30°C.

### Confocal imaging and processing

To test the expression of mCherry-Prp16 under the *GAL7* promoter, both wild-type H99 and *PGAL7*:Prp16 strains were grown in permissive YPG medium overnight and then shifted to either permissive or non-permissive YPD medium. The samples were collected every 3 h for 12 h to observe the level of mCherry-Prp16 signal after transfer into non-permissive media. The cells were pelleted, washed with PBS, and placed on an agarose patch on a microscopic slide. A coverslip was placed on the patch and imaged. The images were acquired by confocal laser scanning microscopy (Zeiss LSM 880) using a 63X oil immersion objective lens, and the images were processed using Zen Blue software and then analysed using ImageJ.

### Semi quantitative RT-PCR and qRT-PCR

Total RNA was isolated from three independent batches of wild-type H99 and *PGAL7*:Prp16 cells grown under Prp16 knockdown conditions (YPD) using triazole reagents, according to the manufacturer’s protocol. Total RNA (20 μg) was subjected to DNase I (NEB) digestion for 40 min, according to the manufacturer’s protocol. Reverse transcription was performed on DNase-treated RNA using 10 µM oligo dT, 10 µM dNTPs and MMLV RT (NEB), according to the manufacturer’s protocol. cDNA (100 ng) was amplified using primers specific to exon-intron-exon sequences. PCR amplicons representing spliced and unspliced transcript segments were resolved on 8% native polyacrylamide gels. The signal intensities for the products were obtained by staining the gel with EtBr, followed by image acquisition using the Bio-Rad gel doc system. Quantification was performed using ImageLab (version 6.1) and normalized to the actin control. For qRT-PCR, the reactions were set up with 20-30ng of cDNA, 250 nM gene-specific primers, and FastStart Universal SYBR Green Master mix (Merck) in a CFX Opus real-time system (BioRad). Fold changes in transcript levels were calculated from the difference in cycle threshold values between Prp16 knockdown and wild type. To obtain the normalized threshold value (ΔΔCt), the ΔCt value was calculated by subtracting the Ct value for the internal control (actin) from the Ct value for each gene of interest. ΔΔCt was calculated by subtracting the wild-type ΔCt value from the ΔCt value obtained for the Prp16 knockdown. The fold-change was calculated as 2^-(ΔΔCt). The primers and their sequences are listed in Supplementary Table S2.

### RNA seq analysis

DNase-treated and purified RNA samples were subjected to next-generation deep-transcriptome sequencing. The sequencing service was outsourced to Molsys Pvt. Ltd Ahmadabad, following the standard kits and protocols recommended by Illumina. Briefly, total mRNA was purified using Ribozero gold, and the library was prepared with TruSeq Stranded Total RNA according to the manufacturer’s protocols. The libraries were sequenced on the Illumina NovaSeq platform, which provided 150bp paired-end reads with 60–80 million reads. The raw files were subjected to quality checks using fastQC. The adapters and low-quality reads were trimmed using fastP. The trimmed files were aligned to *C. neoformans* genome FungiDB-57 using STAR 2.7.3, with default parameters for TwopassMode.

To analyse genome-wide splicing alterations, aligned (*.bam) files from the WT and Prp16 knockdown datasets were processed along with the gene annotation file (GTF) and genome sequence file (*.fasta) using Python script adapted from [20]. These scripted count reads mapped exon-intron junctions (EIJR), intron-exon junctions (IEJR), and exon-exon junctions (CSR). Introns with a total read count < 10 were filtered out from the analysis. These counts were further imported into another Python script [20] to calculate the intron retention level, the intron retention score (IRS), and the difference in IRS between Prp16 knockdown and WT (IRSdiff). As IRS values in WT and Prp16 knockdown followed a normal distribution, a two-sample Z-test was performed with the null hypothesis of zero difference in IRS between WT and Prp16 knockdown for each intron. These outputs (Supplementary Table S3) were used to categorize the introns affected and unaffected classes for further analysis. To visualize intron retention for specific introns in WT and *PGAL7*:Prp16, Sashimi plots were generated using the Python package rmats2Sashimi using aligned (*.bam) files, gene annotation file (GTF), and genome sequence file (*.fasta).

### Western blot

Wild-type and *PGAL7*:Prp16 cells were grown in permissive and non-permissive media for 3–15 hours. The cells were pelleted and washed with PBS. Cell lysates were prepared for western blot using the tricarboxylic acid (TCA) method. Briefly, cells were vortexed with acid-washed glass beads (Cat. No. G8772) in 13% TCA for 30 minutes at room temperature. The lysate was separated from the beads, pelleted at 13,000 rpm for 10 min, and washed with 80% acetone to remove residual TCA. The pellet was air-dried, resuspended in 4X Laemmli buffer (0.02% bromophenol blue, 30% glycerol, 10% SDS, 250 mm Tris-Cl pH 6.8, 5% β-mercaptoethanol), and denatured at 95°C for 5 min. The samples were loaded on 10% SDS PAGE, followed by electrophoresis, and transferred to a PVDF membrane for 2 h at 30 V using a Bio-Rad wet-transfer apparatus. The membrane was blocked with 3% BSA/5% skimmed milk in Tris-buffered saline (TBS), depending on the primary antibody. After blocking, the membrane was incubated with a primary rabbit anti-mCherry antibody (dilution 1:10000) (Abcam, Cat. No. ab213511), overnight at 4°C. The membrane was washed three times with TBST (1X TBS + 0.1% tween 20) and incubated with goat anti-rabbit HRP-conjugated secondary antibody (dilution 1:10,000) for 1 h at room temperature. The blot was washed three times with TBST, and the signals were detected using the chemiluminescence method (Millipore Immobilon Forte HRP substrate).

### Visual data representation

Multiple sequence alignments were performed using Clustal omega. The scatter plots, CDF plots, bar plots, and violin box plots from RNA-seq data were generated with R studio (R version 4.2.1) using the ggplot2 package. The growth curve and bar plot for semi-qRT-PCR quantification were generated using GraphPad Prism (Version 8.4.2). Microsoft PowerPoint 2019 was used to design all schematics and assemble the figures.

### Statistical and reproducibility

Unless otherwise mentioned, all experiments were performed in at least three batches. All plots indicate the standard deviation and mean of independent experiments. Statistical significance was calculated using one-way analysis of variance (ANOVA) with Tukey’s multiple comparison test or unpaired t-test. Statistical significance was set at *p* ≤ 0.05. All analyses were performed using GraphPad prism version 8.4.2. The statistical significance of the plots generated from the RNA-seq data was calculated using the R package ggplot (equivalent to the Wilcoxon rank-sum test). The significances are mentioned as ns, p ≥ 0.05; * - p < 0.05; ** - p < 0.01; *** - p < 0.001; **** - p < 0.001.

## Supplementary Material

Supplemental Material

## Data Availability

This study does not report any original code. This journal follows the Taylor & Francis Share Upon Reasonable Request policy.
