# Supplementary material for: Prp16 enables efficient splicing of introns with diverse exonic consensus elements in the short-intron rich Cryptococcus neoformans transcriptome
Source: RNA Biol. 2025 Mar 10;22(1):1–14. doi: 10.1080/15476286.2025.2477844 (PMC11913375; doi:10.1080/15476286.2025.2477844)
Supplement: Supplemental Material [file KRNB_A_2477844_SM0611.zip › Supplementary information.docx]

**Supplementary information**


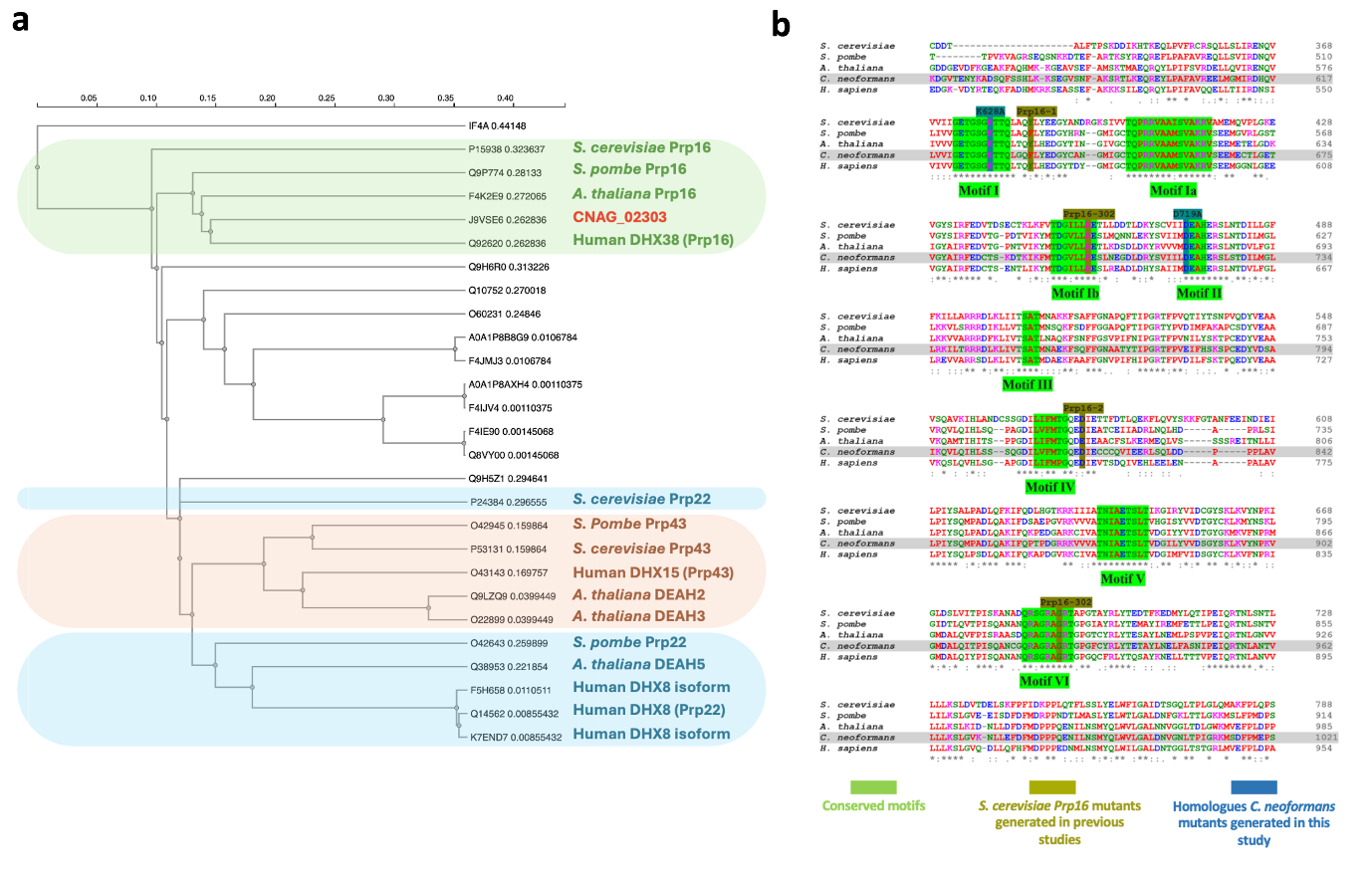


**Supplementary Figure S1.** CNAG_02303 is Prp16 orthologue in C. neoformans. (a) Guide tree from multiple sequence alignment of CNAG_02303 with 25 most closely related homologous proteins from *S. cerevisiae, S. pombe, A. thaliana and H. sapiens* reference proteome. (b) Multiple sequence alignment of C-terminal domain of predicted *C. neoformans* Prp16 (CNAG_02303) with Prp16 known in *S. cerevisiae, S. pombe, A. thaliana and H. sapiens*. Conserved motifs and residues used to generate mutants are highlighted.


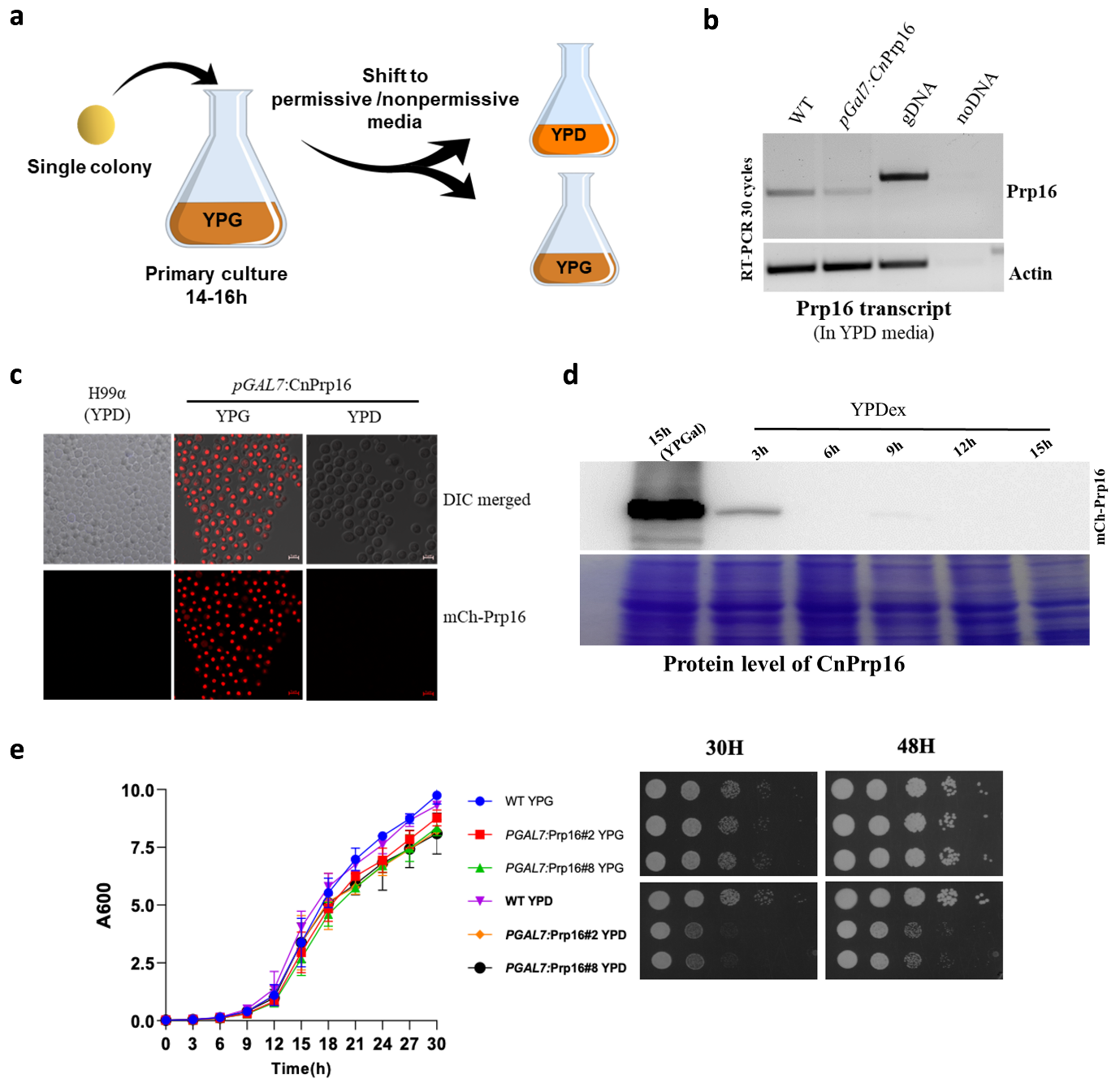


**Supplementary Figure S2.** Validation of *C. neoformans* Prp16 conditional knockdown. (a) Schematics for growth conditions for validation of conditional knockdown of Prp16. (b) Semi-quantitative RT-PCR on RNA isolated from WT and *PGAL7*:Prp16 grown in YPD media for 12-15 hours. (c) Confocal imaging was used to examine the expression of mCherry-Prp16 under the *GAL7* promoter when WT and *PGAL7*:Prp16 cells were grown in YPG and YPD media for 12 hours. (d) Western blot for mCherry-Prp16 when *PGAL7*:Prp16 cells first grown in YPG media and shifted to YPD media for different time duration. (e) Growth profile derived by OD_600_ measurements and also by 10-fold serial dilutions platted on agar plates incubated at 30 ºC for WT and *PGAL7*:Prp16 strains. Both cell types were revived in YPG media and shifted to permissive YPG and nonpermissive YPD media.


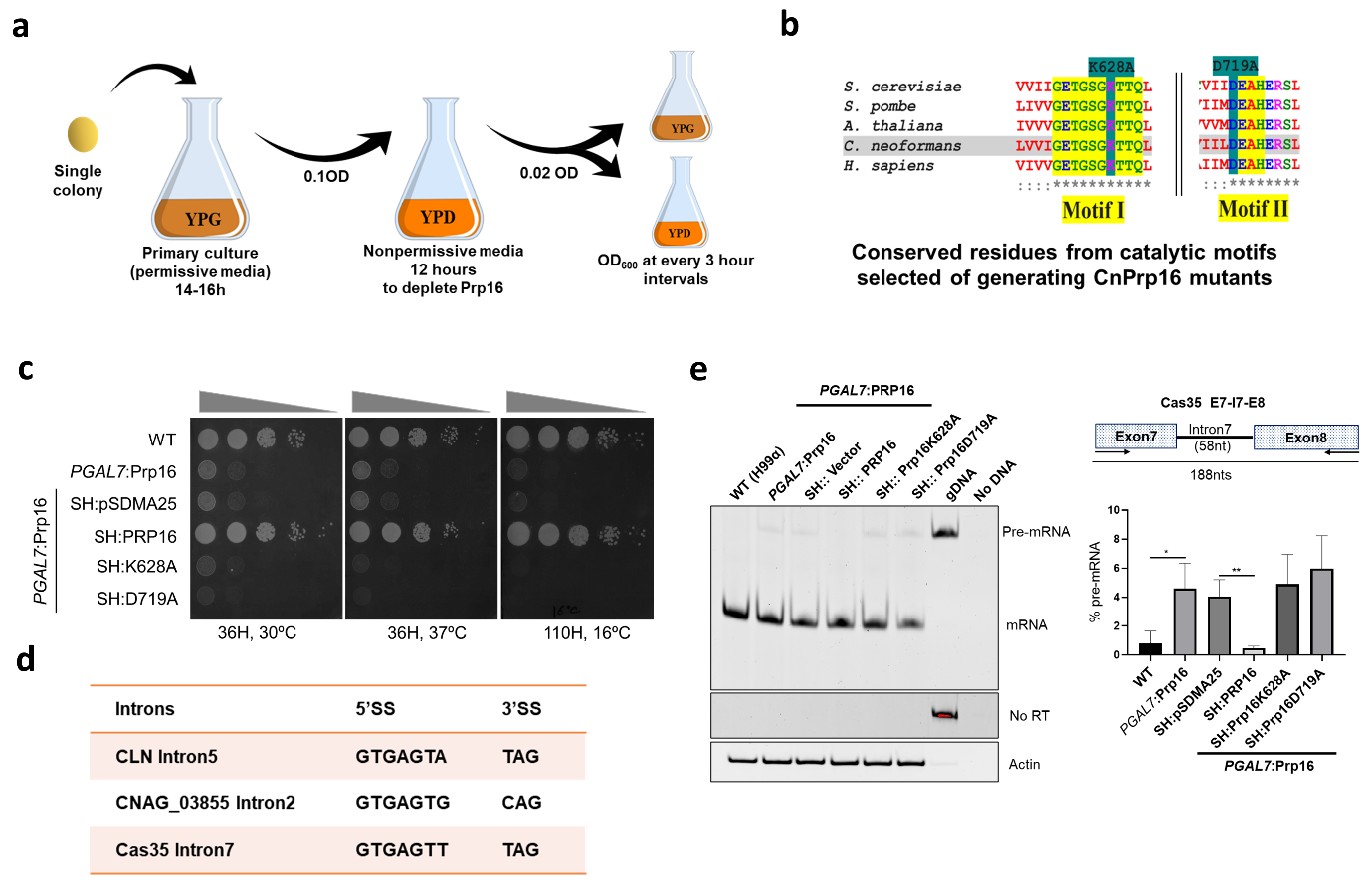


**Supplementary Figure S3.** (a) Schematic depiction of growth conditions for functionally significant knockdown of Prp16. (b) Conserved residues from helicase motif I and motif II chosen for generating Prp16 helicase domain mutants. (c) Growth profile by 10-fold serial dilution in agar plates incubated at 30 ºC, 37ºC and 16ºC for WT, *PGAL7*:Prp16 and *PGAL7*:Prp16 strains expressing either wild type PRP16 (*PGAL7*:Prp16 SH:PRP16) or helicase domain mutants *PGAL7*:Prp16 SH:Prp16^K628A^ and *PGAL7*:Prp16 SH:Prp16 ^D719A^ from safe haven locus. (d) 5’ splice site and 3’ splice site residues of introns chosen for semi-quantitative RT-PCR. (e) Splicing assay of Cas35 intron7by semi-quantitative RT-PCR on RNA isolated from *C. neoformans* WT, *PGAL7*:Prp16 and *PGAL7*:Prp16 strains expressing WT CnPRP16 or the mutants K628A and D719A from safe haven locus. All strains were grown in the condition described previously for Prp16 knockdown. Quantification was done from three biological replicates. One-way ANOVA with Tukey's multiple comparison test was used to calculate significance (ns, P ≥ 0.05; * - P < 0.05; ** - P < 0.01; *** - P < 0.001; **** - P < 0.001).


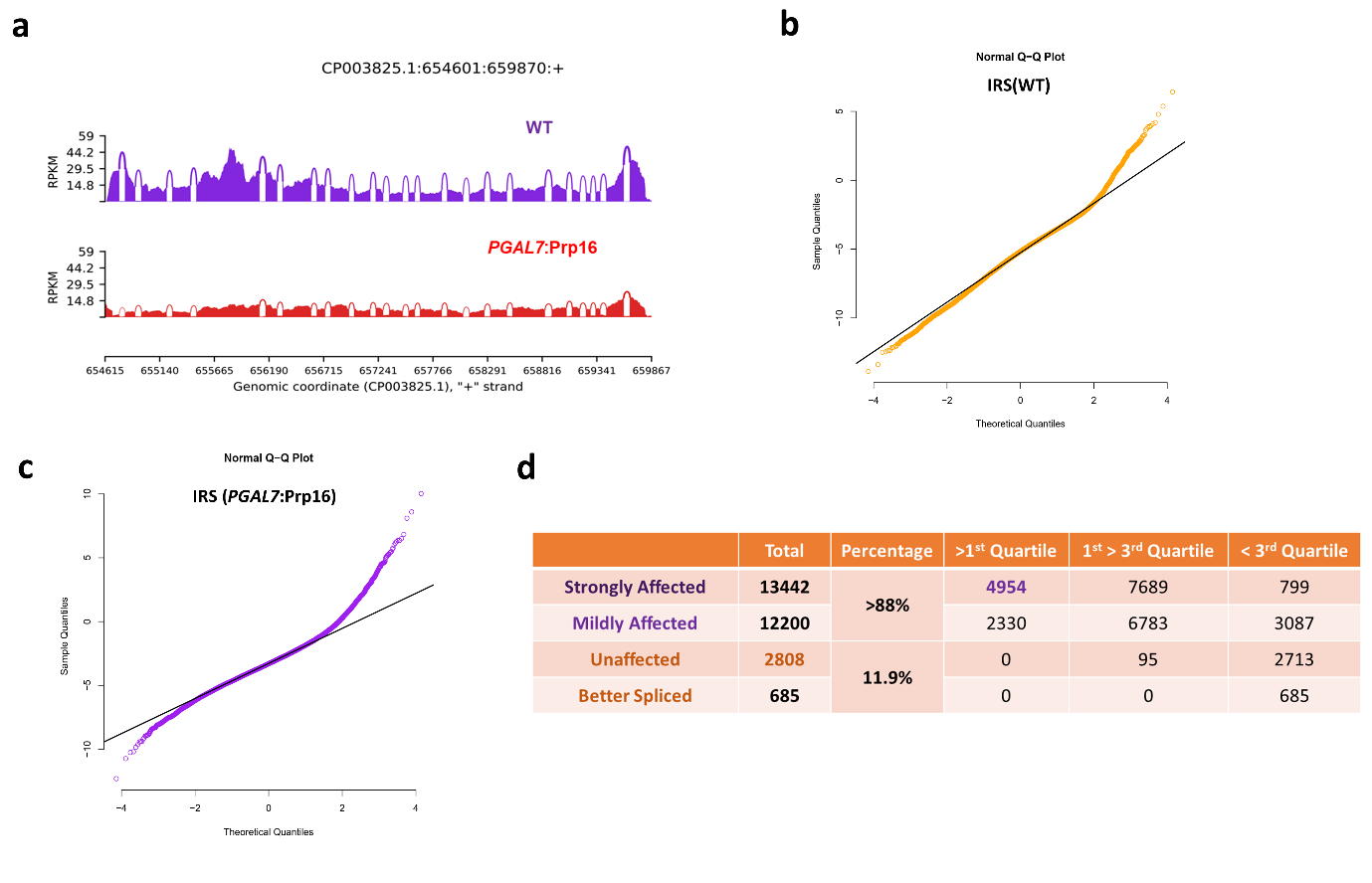


**Supplementary Figure S4.** (a) Normalized reads were obtained from RNA seq of WT and *PGAL7*:Prp16, aligned to Prp16 locus. Decreased reads in *PGAL7*:Prp16 reaffirm transcriptional downregulation of Prp16 via promoter shutdown in YPD media. (b) and (c) Q-Q plot for IRS values in WT and *PGAL7*:Prp16, respectively. (d) In genome-wide splicing analysis, numbers of introns were found to be strongly affected, mildly affected, and unaffected. More than 88% of the introns are affected on Prp16 knockdown in *PGAL7*:Prp16 strain.


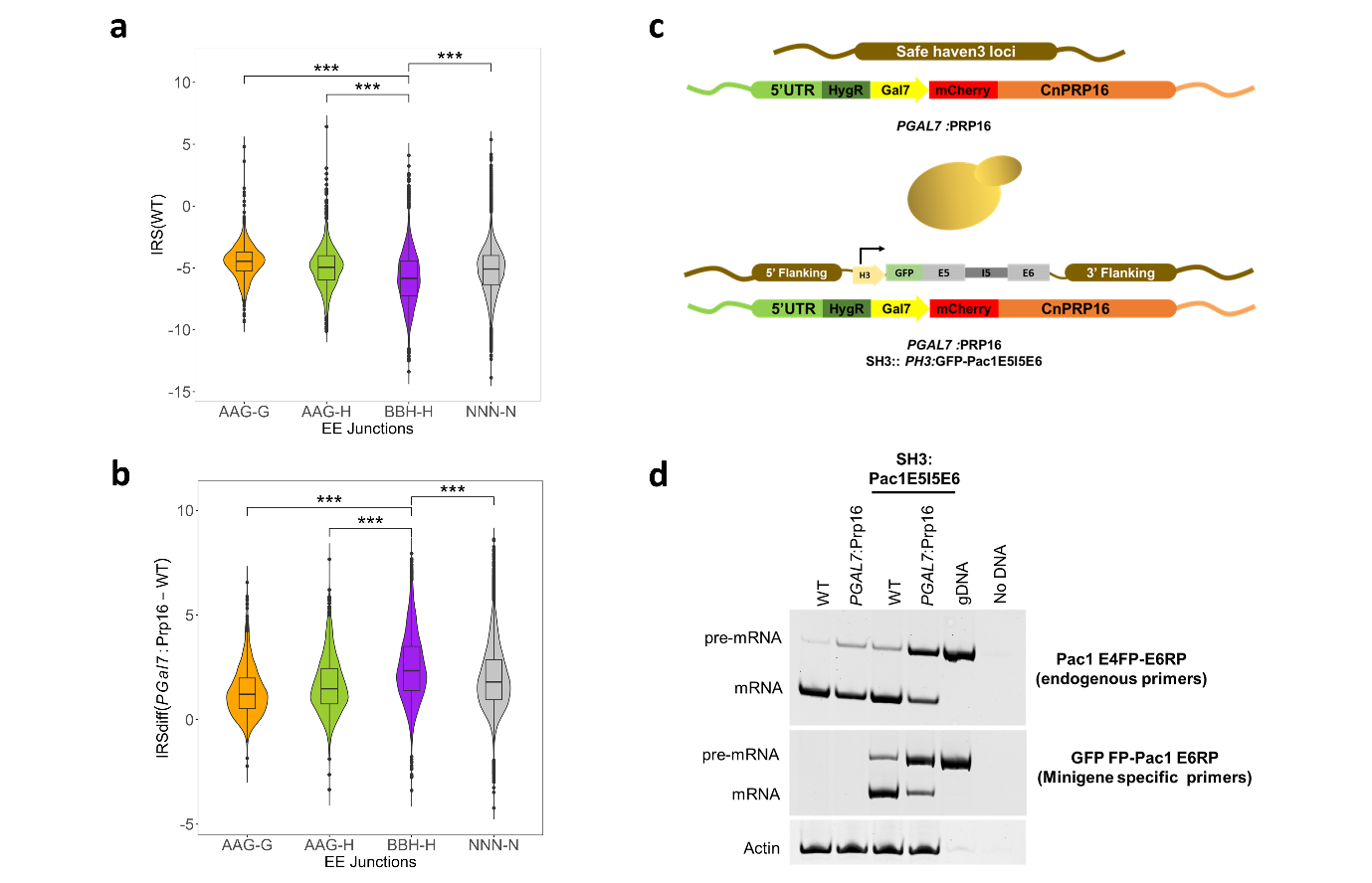


**Supplementary Figure S5.** (a) Violin-box plot of IRS in WT for each group of classified introns based on their last three nucleotides of 5'exon and 1^st^ nucleotide of 3'exon. (b) Violin-box plot of IRS differences between *PGAL7*:Prp16 and WT for each group of classified introns based on their last three nucleotides of 5'exon and 1^st^ nucleotide of 3'exon. Asterisks indicate statistically significant differences, as determined by the Wilcoxon rank-sum test within the R package ggplot2. (c) Schematic depiction of strain with GFP-Pac1E5I5E5 minigene under *H3* promoter at safe haven 3 locus. (d) Semi-quantitative RT-PCR for Pac1 E5I5E6 in WT, *PGAL7*:Prp16 strains with *PH3*:GFP-Pac1E5I5E6 integrated at safe haven 3 loci. RT-PCR was done with endogenous primers (which detect cDNA from both endogenous and minigene expressed transcripts) and minigene-specific primers. No amplicon from untransformed WT and *PGAL7*:Prp16 in the case of minigene-specific primers confirms accurate detection of mini-transcript expressed from safe haven 3 locus.


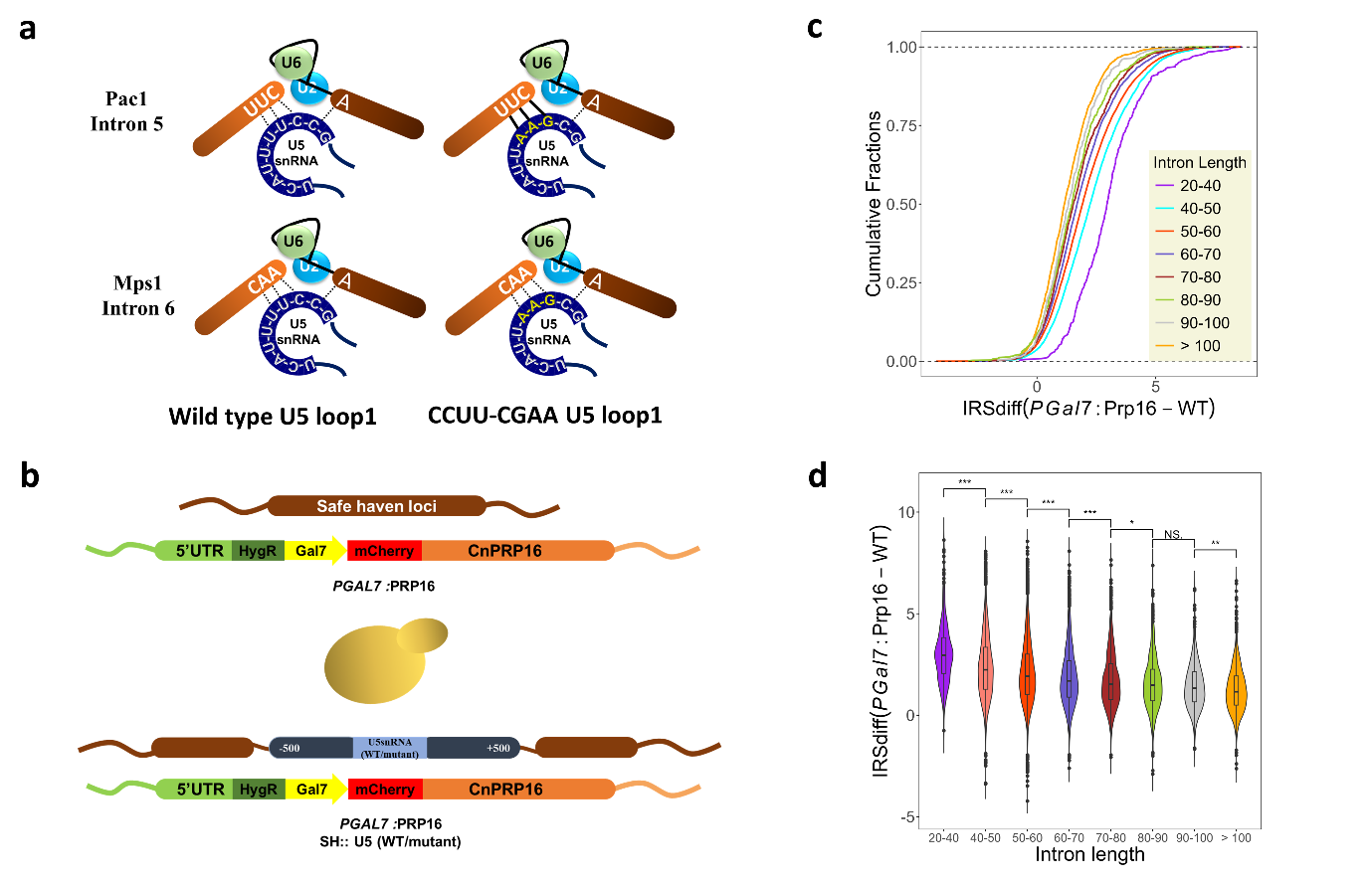


**Supplementary Figure S6.** (a) Schematic depiction for pre-mRNA interaction with WT U5 and loop1 mutant U5^CUU-GAA^ for Pac1E5I5E6 and Mps1E6I6E7. (b) Schematic depiction of strain with WT U5 and loop1 mutant U5^CUU-GAA^ expressed from safe haven locus. (c) CDF and (d) Violin-box plot of IRS differences between *PGAL7*:Prp16 and WT for each group of classified introns based on the size range of 10nts difference

**Supplementary Table S1.** Strains used in this study.

*C. neoformans* Strains used in this study

| Strains | Genotype | Reference |
| --- | --- | --- |
| H99α | Wild type | ((Perfect et al., 1993)) |
| *PGAL7*:Prp16 | MATα, PRP16:: HygB-GAL7p-mCherry-Prp16 | This study |
| *PGAL7*:Prp16, SH:PRP16 | MATα, PRP16:: HygB-*PGAL7*-mCherry:Prp16, SH::PRP16-NAT (pSDMA25) | This study |
| *PGAL7*:Prp16, SH::K628A | MATα, PRP16:: HygB-*PGAL7*-mCherry:Prp16, SH::Prp16 K628A-NAT (pSDMA25) | This study |
| *PGAL7*:Prp16,  SH:: D719A | MATα, PRP16::HygB-GAL7p-mCherry-Prp16, SH::PRP16 D719A-NAT (pSDMA25) | This study |
| H99α, SH3::Pac1I5 | MATα, SH3:: *PH3*-GFP:Pac1E5-I5-E6-NAT (pEE27) | This study |
| H99α, SH3::Pac1I5 AAG | MATα, SH3:: *PH3*-GFP:Pac1E5-I5-E6(5’SS TTC-AAG)-NAT (pEE27) | This study |
| H99α, SH3::Pac1I5  AAG-G | MATα, SH3:: *PH3*-GFP:Pac1E5-I5-E6(5’SS-3’SS TTCA-AAGG)-NAT (pEE27) | This study |
| *PGAL7*:Prp16, SH::Pac1I5 | MATα, Prp16:: HygB-GAL7p-mCherry-Prp16, SH3:: *PH3*-GFP:Pac1E5-I5-E6-NAT (pEE27) | This study |
| *PGAL7*:Prp16,  SH::Pac1I5 AAG | MATα, Prp16:: HygB-GAL7p-mCherry-Prp16, SH3:: *PH3*-GFP:Pac1E5-I5-E6(5’SS TTC-AAG)-NAT (pEE27) | This study |
| *PGAL7*:Prp16,  SH::Pac1I5 AAG-G | MATα, Prp16:: HygB-GAL7p-mCherry-Prp16, SH3:: *PH3*-GFP:Pac1E5-I5-E6(5’SS,3’SS TTCA-AAGG)-NAT (pEE27) | This study |
| *PGAL7*:Prp16,  SH::U5 | MATα, PRP16:: HygB-*PGAL7*-mCherry:Prp16, SH::U5 -NAT (pSDMA57) | This study |
| *PGAL7*:Prp16,  SH::U5 CUU-GAA | MATα, PRP16:: HygB-*PGAL7*-mCherry:Prp16, SH:: U5 CUU-GAA -NAT (pSDMA57) | This study |

Perfect, J. R., Ketabchi, N., Cox, G. M., Ingram, C. W., & Beiser, C. L. (1993). Karyotyping of Cryptococcus neoformans as an epidemiological tool. *Journal of Clinical Microbiology*, *31*(12), 3305–3309. <https://doi.org/10.1128/jcm.31.12.3305-3309.1993>

**Supplementary Table S2.** Sequences of primers and oligos used in this study.

Primers/oligos used in this study

| SN | Primer name | Sequence | Description |
| --- | --- | --- | --- |
| 1 | Prp16 H99 5’UTR FP SACI | TCA GAG CTC TGATTCTCTTCATTCTCCT | Amplification of 1kb upstream fragment from ATG of *C. neoformans* Prp16 |
| 2 | Prp16 H99 5’UTR RP SACI | TCAGAGCTCGTTCAGGTATGTAGAGGATGA |  |
| 3 | H99 PRP16 P1 HINDIII | TCCAAGCTTATGTCCGCCAGGTCACCAA | Amplification of 1kb downstream fragment from ATG of *C. neoformans* Prp16 |
| 4 | H99 PRP16 P2 XHOI | ATGCTCGAGTTCATTCCATCTGTCCCTGTTT |  |
| 5 | mCherry FP | AACATCAAGTTGGACATCACCTCCCA | mCherry FP for locus specific PCR validation of Gal7:mCherry-Prp16 |
| 6 | Prp16 (4350) RP | ACTCTTTAGCGTTGAGATCC | Used with mCherry FP for locus specific PCR validation of Gal7:mCherry-Prp16 |
| 7 | Prp16 (176 CDS) RP | GTCGAAACAGCTCTAAGGAATGC | Used with H99 PRP16 P1 HINDIII for qRT-PCR of Prp16 |
| 8 | CnH99Prp16RP (XbaI) | TTGTCTAGATTAAATGCCTCCTGCTCGTC | Used with H99 PRP16 P1 HINDIII for amplification of full length gene body of Prp16 or cDNA |
| 9 | CnPrp16-500FP(XhoI) | GTACCGGGCCCCCCCTCGAGAACGTAGAATACCGCGAAATCG | Used to clone Prp16 loci in safe haven plasmid |
| 10 | CnPrp16+500RP(PstI) | GTGGATCCCCCGGGCTGCAGATTGAAGAAGAACCAGTTCAGC |  |
| 11 | cnPrp16_K628A_FP | GGGTCAGGCGCAACAACTCAG | For K628A site directed mutagenesis of Prp16 |
| 12 | cnPrp16_K628A_RP | CTGAGTTGTTGCGCCTGACCC |  |
| 13 | cnPrp16_D719A_FP | ATCATCCTTGCTGAAGCACAC | For D719A site directed mutagenesis of Prp16 |
| 14 | cnPrp16_D719A_RP | GTGTGCTTCAGCAAGGATGAT |  |
| 15 | MPS1I6_FP | CGTTCATACAGATTTGAAGCC | RT-PCR OF Mps1 E6I6E7 |
| 16 | MPS1I6_RP | CTTCAATACCTTCTGATTGTTC |  |
| 17 | 3855I2_FP | GGAACTCTCTACGAGATGGG | RT-PCR OF CNAG_03855 E2I2E3 |
| 18 | 3855I2_RP | GTATGGCGAAAAGTTGTGTCG |  |
| 20 | SEC72I4_FP | CAATACGAGGACGCAAAGC | RT-PCR OF Sec72 E4I4E5 |
| 21 | SEC72I4_RP | TCATTGAACGGTGTCTGGG |  |
| 22 | CAS35I7_FP | AGAGGTGGAGGATGTTATTGC | RT-PCR OF Cas35 E7I7E8 |
| 23 | CAS35I7_RP | ATTCCAAGAATCTTGAAAGGCG |  |
| 24 | 2654I4_FP | GACGAAAATAGAGCATGGGG | RT-PCR OF DNA ploξ E4I4E5 |
| 25 | 2654I4_RP | TCATAAGCCATAACGACTGG |  |
| 26 | 00649I3_FP | GCCGACATCATCGAACTCG | RT-PCR OF CNAG_00649 E3I3E4 |
| 27 | 00649I3_RP | CCATGAACAAGACGGGAGC |  |
| 29 | RPC1I11_FP | TGGAGGTATCGTGCAGTTCC | RT-PCR OF RPC1 E11-I11-E12 |
| 30 | RPC1I11_RP | TGGGAGGAACAAAAGACTCG |  |
| 31 | SH FP1 | GGGTATGCCACAGATGCAGAT | Used for validation of integration at safe haven loci |
| 32 | SH RP1 | ACTGGTGAGTACTCAACCAAG |  |
| 33 | SH FP2 | TCAGCAACGCCGTTGAATCCT |  |
| 34 | SH RP2 | TTGGATCCTCAATTGTCTCCT |  |
| 35 | SH3FP | TCTACGTTGGCGCTTCAAGC | Used with SH FP2 and SH RP1 for validation of integration at safe haven 3 loci |
| 36 | SH3RP | TTGGAGTCAACAGCCGTGGG |  |
| 37 | U5_up_FP | CTGCAGTCTGCTGACGAACTCTATTC | Used for amplification and cloning of U5 snRNA with 500bp upstream and downstream sequence |
| 38 | U5_down_RP | CTCGAGGTACAGGAATAGACTCGTCG |  |
| 39 | Pac1I5(5PSS-AAG)FP | CTACGGACAAAGGTACGTCGT | 5’SS TTC-AAG mutagenesis of pac1 E5I5E6  3’SS C-G mutagenesis of pac1 E5I5E6 |
| 40 | Pac1I5(5PSS-AAG)RP | ACGACGTACCTTTGTCCGTAG |  |
| 41 | Pac1I5(5PSS-AAG)FP | CTACGGACAAAGGTACGTCGT |  |
| 42 | Pac1I5(5PSS-AAG)RP | ACGACGTACCTTTGTCCGTAG |  |
| 43 | U5-CUU_FP | CGAATAAATCTCTCGCGAATTACTAGAGATATCC | Used for Site directed mutagenesis of U5 loop1 CCU-GGA |
| 44 | U5-CUU_RP | GGATATCTCTAGTAATTCGCGAGAGATTTATTCG |  |
